# Timely Albumin Improves Survival in Patients With Cirrhosis on Diuretic Therapy Who Develop Acute Kidney Injury: Real-World Evidence in the United States

**DOI:** 10.1016/j.gastha.2022.10.008

**Published:** 2022-10-26

**Authors:** Ray W. Kim, Karthik Raghunathan, Greg S. Martin, E. Anne Davis, Navreet S. Sindhwani, Santosh Telang, Kunal Lodaya

**Affiliations:** 1 Division of Gastroenterology and Hepatology, Stanford University, Stanford, California; 2 Department of Anesthesiology, Duke University, Durham, North Carolina; 3 Department of Medicine, Emory University, Atlanta, Georgia; 4 Grifols Shared Services North America (SSNA), Research Triangle Park, North Carolina; 5 Boston Strategic Partners, Inc, Boston, Massachusetts

**Keywords:** Length of Stay, In-Hospital Mortality, Decompensation, AKI

## Abstract

**Background and Aims:**

Patients admitted with decompensated cirrhosis who develop acute kidney injury (AKI) tend to experience poor outcomes, even if provided with increased organ support such as renal replacement therapies. We assessed the association of albumin administered ≤24 hours of admission with hospital length of stay (LOS) and in-hospital mortality.

**Methods:**

The Cerner Health Facts Database was queried for hospitalized patients with cirrhosis who had >0.3 mg/dL increase in serum creatinine within 48 hours and received diuretics following admission between January 2009 and April 2018. This study received institutional review board exemption through federal regulation 45CFR46. Albumin infusion was “timely” if administered ≤24 hours after admission and “nontimely” if administered >24 hours after admission or not at all. Two subgroups were assessed: the AKI_LOS_ subgroup (patients who survived to discharge) and the AKI_MORTALITY RISK_ subgroup (patients with the highest risk of mortality, ie, AKI stage 3).

**Results:**

A total of 4135 hospitalizations with cirrhosis and AKI were grouped into AKI_LOS_ (n = 3321) and AKI_MORTALITY RISK_ (n = 609) subgroups. Albumin administration occurred in 59.7% of the AKI_LOS_ subgroup and 77.8% of the AKI_MORTALITY RISK_ subgroup, but timely treatment only occurred in 25.9% and 35.8% of encounters within these subgroups, respectively. Risk-adjusted analysis showed timely albumin administration to be associated with a 15.5% reduction (*P* < .01) in LOS in the AKI_LOS_ subgroup and a 49% reduction in the odds of death (adjusted odds ratio: 0.51; *P* < .01) in the AKI_MORTALITY RISK_ subgroup, when compared to the nontimely group.

**Conclusion:**

Among patients with cirrhosis and AKI, treatment with albumin ≤24 hours after admission was associated with a shorter LOS and lower risk of death in patients with stage 3 AKI.

## Introduction

Cirrhosis is the final common pathway by which progressive fibrosis in various chronic liver diseases leads to portal hypertension and liver failure. Cirrhosis is a common cause of premature death. According to the Global Burden of Disease estimate of 2010, cirrhosis caused 49,500 deaths and loss of 1.2 million years of life in the United States, which made it the 8th most important cause of premature deaths.^1^ Patients with decompensated cirrhosis and end-stage liver disease (ESLD) often require hospital-based care for a number of complications that frequently lead to mortality despite intensive care with organ support. Patients that do survive are frequently rehospitalized within 30 days, ultimately placing substantial financial burden on the health-care delivery system without meaningful improvement in their quality of life.^2^, ^3^, ^4^ The total annual cost of hospitalization for patients with decompensated cirrhosis was estimated to increase from $7 billion in 2005 to over $16 billion in 2015.^5^^,^^6^

Acute kidney injury (AKI) is a common complication among patients hospitalized with decompensated cirrhosis.^7^ It is diagnosed in 20%–50% of such patients^8^, ^9^, ^10^, ^11^, ^12^ and is associated with increased mortality.^13^, ^14^, ^15^, ^16^, ^17^, ^18^, ^19^, ^20^ Given the complexity of care delivered in this setting including management of precipitating factors, plasma volume expansion and electrolyte replacement, and administration of vasopressors, increased health-care expenditure is not surprising.^21^^,^^22^ A 2019 study using data from the Nationwide Inpatient Sample in the United States found that hospitalizations with cirrhosis and AKI have significantly greater hospital length of stay (LOS) and median hospital charges, as well as mortality rates, when compared to those without AKI.^23^ Early identification of AKI with treatment is warranted to reduce mortality, hospital LOS, as well as cost of care.^24^, ^25^, ^26^

Treatment with albumin may play a critical role in the management of patients with decompensated cirrhosis and AKI.^27^, ^28^, ^29^, ^30^, ^31^, ^32^ Albumin improves outcomes in patients with decompensated cirrhosis undergoing large-volume paracentesis and in patients being treated for spontaneous bacterial peritonitis (SBP). Despite unambiguous evidence, albumin may not be used perhaps in part to its higher cost vs crystalloids. In this study, we use a nationwide electronic health record database to study patterns of treatment with albumin in patients hospitalized with cirrhosis and AKI and to also examine the association of early administration of albumin with hospital LOS and in-hospital mortality.

## Materials and Methods

The Western Institutional Review Board (Puyallup, WA) determined that this study was exempt from the informed consent requirement. Approval was obtained after formulation of a predefined statistical analysis plan.

### Data Source

We extracted data from the Cerner Health Facts database (Cerner Corp, Kansas City, MO) which contains deidentified electronic health records data from >700 participating clinical facilities across the United States.

### Patient Population

We identified adults (≥18 years old) hospitalized between January 1, 2009, and April 30, 2018, with a diagnosis of cirrhosis using the International Classification of Disease nineth and tenth revision codes and current procedural terminology codes.^33^ We then used the following criteria to identify cases where patients developed AKI while having received diuretics within 30 days prior to admission: serum creatinine (SCr) rising >0.3 mg/dL compared to the baseline within 48 hours.

We grouped patients with AKI as per the Kidney Disease Improving Global Outcomes guidelines framework^34^ into stages 1, 2, and 3. The baseline SCr used to determine staging was the value closest to hospital admission within a 90-day lookback period (Logical Observation Identifiers Names and Codes, or LOINC shown in Table A2).^35^^,^^36^ If these data were unavailable, we used the first SCr recorded during the index hospitalization. Urine output data were not used for staging as they were unavailable. We defined diuretic therapy based on furosemide or spironolactone receipt. If patients had multiple encounters/hospitalizations, we included their first encounter after January 2009.

We created 2 subgroups of encounters that were not mutually exclusive; an encounter could be included in both subgroups. The AKI_LOS_ subgroup comprised patients who were hospitalized and survived to discharge. We excluded decedents because their LOS was truncated by death. The AKI_MORTALITY RISK_ subgroup comprised patients with the highest risk of fatality, namely those with AKI stage 3, as AKI stage 1 and 2 do not portend mortality in the same way as stage 3.^14^ Organ support with renal replacement therapy (RRT) was also considered as stage 3 AKI.

### Exposures, Outcomes, and Covariates

In both the AKI_LOS_ and AKI_MORTALITY RISK_ subgroups, we categorized patients based on exposure to albumin, defined by the receipt of any intravenous fluid preparation containing albumin, as either receiving timely treatment (administration ≤24 hours of admission) or not (albumin was administered >24 hours after admission or not at all).

In the AKI_LOS_ subgroup, we evaluated LOS as the key outcome (the period from hospital admission to discharge in hours). In the AKI_MORTALITY RISK_ subgroup, all-cause in-hospital fatality was identified by the discharge status “deceased”.

To account for baseline differences between the exposure categories, we extracted information on patient characteristics such as age group, sex, ethnicity, admission type, payer, year of admission, and hospital characteristics such as bed size, teaching status, urban vs rural setting, acute vs nonacute hospitals, and census region. Acuity of illness was measured based on mechanical ventilation, receipt of antibiotics, and nonselective beta-blockers. Patients’ blood glucose levels (Table A2) and model for end-stage liver disease (MELD-Na) scores (calculated at presentation using laboratory data) were also considered to ensure that differences in baseline severity of illness were accounted for when comparing outcomes in patients exposed vs not exposed to timely treatment with albumin.^37^, ^38^, ^39^

### Statistical Analyses

Descriptive statistics were calculated for patient and hospital characteristics and summarized via counts and percentages for binary or categorical variables and with means and standard deviations (SDs) for continuous variables.

In the AKI_LOS_ subgroup, the association of timely albumin administration with the continuous outcome variable LOS was examined using the generalized linear model with gamma distribution and a logarithmic link function (as the distribution of LOS was skewed). In the AKI_MORTALITY RISK_ subgroup, the association of timely albumin administration with in-hospital death was examined using a multivariable logistic regression model. In both the generalized linear model and logistic regression models, generalized estimating equations with robust standard errors were used to take clustering at the hospital level into consideration (ie, to account for within-hospital correlations among patients). Adjusted parameter estimates and adjusted odds ratios, for LOS and mortality, respectively, along with confidence intervals (CIs) were calculated.

The covariates considered in both multivariable analyses are listed in Tables 1 and 2, including patient and hospital characteristics; receipt of mechanical ventilation, antibiotics, and nonselective beta-blockers; MELD-Na scores at presentation; and patients’ blood glucose levels.^37^, ^38^, ^39^ We also examined important complications including rates of gastrointestinal (GI) bleeding, hepatic encephalopathy, and SBP (Table A1). All statistical analyses were performed in SAS version 9.4 (SAS Institute Inc, Cary, NC).

**Table 1:** Patient Characteristics^a^

Characteristics	All AKI (N = 4135)	AKI_LOS_ (N = 3231)	AKI_MORTALITY RISK_ (N = 609)
Age (mean ± SD)	59.8 ± 12.2	59.5 ± 12.2	59 ± 12.5
Age-group, n (%)			
18–29	41 (1.0)	30 (0.9)	9 (1.5)
30–49	709 (17.2)	571 (17.7)	110 (18.1)
50–64	1998 (48.3)	1584 (49.0)	278 (45.7)
65+	1387 (33.5)	1046 (32.4)	212 (34.8)
Female, n (%)	1684 (40.7)	1306 (40.4)	243 (39.9)
Ethnicity, n (%)			
Caucasian	3094 (74.8)	2433 (75.3)	425 (69.8)
African American	433 (10.5)	346 (10.7)	70 (11.5)
Hispanic	69 (1.7)	54 (1.67)	9 (1.5)
Asian/Pacific Islander	44 (1.1)	30 (0.93)	11 (1.8)
Other^b^	439 (10.6)	416 (12.9)	83 (13.6)
Not specified^c^	56 (1.4)	36 (1.1)	11 (1.8)
Admission type, n (%)			
Emergency	3548 (85.8)	2776 (85.9)	529 (86.9)
Urgent	367 (8.9)	277 (8.6)	46 (7.6)
Elective	220 (5.3)	178 (5.5)	34 (5.6)
Payer group, n (%)			
Medicare	1678 (40.6)	1315 (40.7)	252 (41.38)
Medicaid	873 (21.1)	708 (21.9)	119 (19.5)
Commercial	844 (20.4)	657 (20.3)	126 (20.7)
Other^d^	179 (4.3)	128 (4.0)	24 (3.9)
Self	186 (4.5)	150 (4.6)	25 (4.1)
Not specified	375 (9.1)	273 (8.5)	63 (10.3)
Baseline clinical parameters, mean ± SD			
Charlson comorbidity Index	8.4 ± 3.6	8.4 ± 3.6	9.0 ± 3.8
MELD-Na	21.2 ± 8.7	20.3 ± 8.2	25.7 ± 9.0
Hyperglycemia, n (%)	1452 (35.1)	1123 (34.8)	208 (34.2)
Hypoglycemia, n (%)	194 (4.7)	101 (3.1)	53 (8.7)
Both hypoglycemia and hyperglycemia, n (%)	527 (12.7)	326 (10.1)	115 (18.9)
Neither hypoglycemia nor hyperglycemia, n (%)	1962 (47.5)	1681 (52.0)	233 (38.3)
Comorbidities, n (%)			
Spontaneous bacterial peritonitis, n (%)	425 (10.3)	291 (9.0)	78 (12.8)
Gastrointestinal bleeding, n (%)	634 (15.3)	416 (12.9)	92 (15.1)
Hepatic encephalopathy, n (%)	1518 (36.7)	1124 (34.8)	224 (36.8)

a Remaining patient and hospital characteristics available in Tables A3 and A4.

b Includes Native American, Biracial, Mid-Eastern Indian.

c Includes null, not mapped, unknown.

d Includes government, military, nongovernmental organization, and work compensation payer groups.

**Table 2:** Patient Characteristics by AKI Subgroups^a^

Characteristics	AKI_LOS_ (n = 3231)		AKI_MORTALITY RISK_ (n = 609)	
	Timely (n = 838)	Nontimely (n = 2393)	Timely (n = 218)	Nontimely (n = 391)
Age, mean ± SD	57.5 ± 11.3	60.2 ± 12.4	59.1 ± 12.0	59 ± 12.8
Age-group, n (%)				
18–29	8 (1.0)	22 (0.9)	1 (0.5)	8 (2.1)
30–49	162 (19.3)	409 (17.1)	39 (17.9)	71 (18.2)
50–64	469 (56.0)	1115 (46.6)	104 (47.7)	174 (44.5)
65+	199 (23.8)	847 (35.4)	74 (33.9)	138 (35.3)
Female, n (%)	310 (37.0)	996 (41.6)	76 (34.9)	167 (42.7)
Ethnicity, n (%)				
Caucasian	627 (74.8)	1806 (75.5)	152 (69.7)	273 (69.8)
African American	65 (7.8)	281 (11.7)	18 (8.3)	52 (13.3)
Hispanic	14 (1.67)	40 (1.67)	5 (2.3)	4 (1.0)
Asian/Pacific Islander	10 (1.2)	20 (0.84)	6 (2.8)	5 (1.3)
Other^b^	136 (16.2)	280 (11.7)	34 (15.6)	49 (12.5)
Not specified^c^	10 (1.2)	26 (1.1)	3 (1.4)	8 (2.1)
Admission type, n (%)				
Emergency	690 (82.3)	2086 (87.2)	190 (87.2)	339 (86.7)
Urgent	98 (11.7)	179 (7.5)	18 (8.3)	28 (7.2)
Elective	50 (6.0)	128 (5.4)	10 (4.6)	24 (6.1)
Payer group, n (%)				
Medicare	285 (34.0)	1030 (43.0)	78 (35.8)	174 (44.5)
Medicaid	190 (22.7)	518 (21.7)	41 (18.8)	78 (20.0)
Commercial	220 (26.3)	437 (18.3)	55 (25.2)	71 (18.2)
Other^d^	41 (4.9)	87 (3.6)	12 (5.5)	12 (3.1)
Self	38 (4.5)	112 (4.7)	10 (4.6)	15 (3.8)
Null	64 (7.6)	209 (8.7)	22 (10.1)	41 (10.5)
Baseline clinical parameters, mean ± SD				
Charlson comorbidity Index	8.1 ± 3.5	8.5 ± 3.6	9.2 ± 4.0	8.9 ± 3.8
MELD-Na	22.8 ± 8.4	19.4 ± 7.9	29.5 ± 8.1	23.6 ± 8.9
Bilirubin, mg/dL	7.0 ± 8.7	5.3 ± 7.3	11.0 ± 12.2	8.5 ± 11.4
International normalized ratio	2.0 ± 1.1	2.0 ± 1.4	2.7 ± 2.1	2.9 ± 2.7
Creatinine, mg/dL	2.7 ± 1.9	2.3 ± 1.7	4.6 ± 2.3	4.3 ± 2.6
Sodium, mEq/L	138.4 ± 6.3	138.9 ± 6.2	138.5 ± 6.9	139.5 ± 7.7
Hyperglycemia, n (%)	270 (32.2)	853 (35.7)	76 (34.9)	132 (33.8)
Hypoglycemia, n (%)	30 (3.6)	71 (3.0)	23 (10.6)	30 (7.7)
Both hypoglycemia and hyperglycemia, n (%)	73 (8.7)	253 (10.6)	36 (16.5)	79 (20.2)
Neither hypoglycemia nor hyperglycemia, n (%)	465 (55.5)	1216 (50.8)	83 (38.1)	150 (38.4)
Process variables, n (%)				
Mechanical ventilation	74 (8.8)	178 (7.4)	57 (26.2)	103 (26.3)
Vasopressors^e^	128 (15.3)	391 (16.3)	71 (32.6)	151 (38.6)
Antibiotics	673 (80.3)	1862 (77.8)	188 (86.2)	330 (84.4)
Steroids	80 (9.6)	271 (11.3)	21 (9.6)	49 (12.5)
Nonselective beta-blockers	266 (31.7)	899 (37.6)	60 (27.5)	127 (32.5)
Renal replacement therapy	90 (10.7)	123 (5.1)	120 (55.1)	198 (50.6)
Comorbidities, n (%)				
Spontaneous bacterial peritonitis	147 (17.5)	144 (6.0)	39 (17.9)	39 (10.0)
Gastrointestinal bleeding	89 (10.6)	327 (13.7)	34 (15.6)	58 (14.8)
Hepatic encephalopathy	332 (39.6)	792 (33.1)	99 (45.4)	125 (32.0)

Numbers are presented as either n (%) or mean ± standard deviation (SD).

a Remaining patient and hospital characteristics available in Tables A3 and A4.

b Includes Native American, Biracial, Mid-Eastern Indian.

c Includes null, not mapped, unknown.

d Includes government, military, nongovernmental organization, and work compensation payer groups.

e Vasopressors include dobutamine, dopamine, epinephrine, norepinephrine, phenylephrine, vasopressin.

## Results

### Study Participants

**Figure 1:**
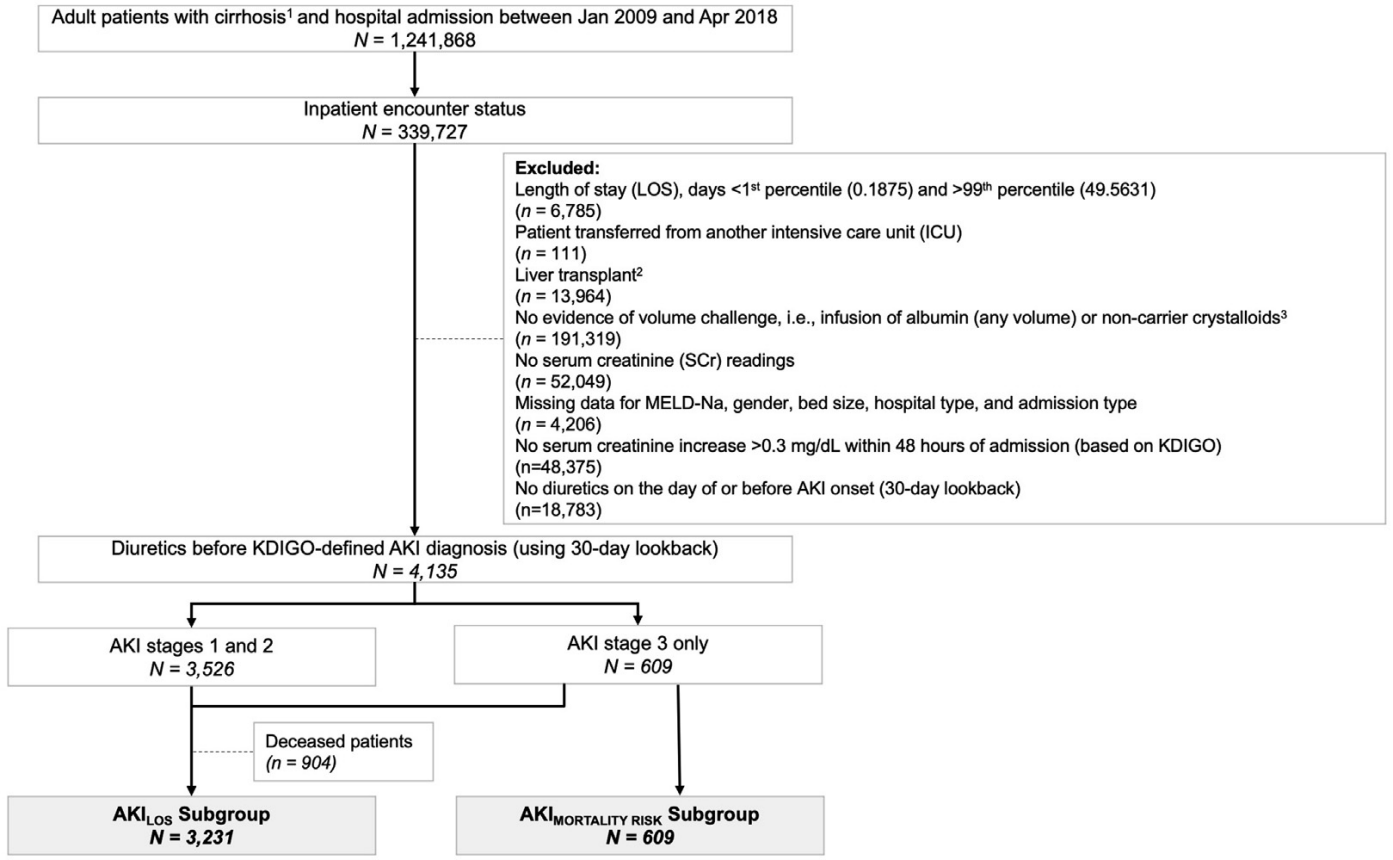
Consort diagram for patient selection. Analysis conducted at visit level. ^1^Using cluster variables for cirrhosis (except category C7) from Lu M et al. Clin Epidemiol. 2017;9:369–376. ^2^Category C1 from Lu M et al. Clin Epidemiol. 2017;9:369–376. ^3^Crystalloid administration as any type of intravenous crystalloid infusion used for volume expansion with at least 1 order delivered in a container >250 ml (ie, 500-ml or 1000-ml bags) to exclude potential carriers (eg, for vasoactive or sedative drugs). KDIGO, Kidney Disease Improving Global Outcomes; MELD-Na, model for end-stage liver disease.

The Cerner database contained 339,727 records of adults (≥18 years old) hospitalized with cirrhosis during the study period, of which 257,332 were excluded because of (1) liver transplantation (n = 13,964), (2) no measures of intravenous volume expansion (n = 191,319), or (3) no SCr data (n = 52,049). Finally, 6896 records were excluded if the LOS was <1st percentile (0.19 days) or >99th percentile (49.56 days) (n = 6785) or if they involved an interfacility transfer (n = 111). Among the 75,499 remaining encounters, 4135 (5.5%) met the inclusion criteria for AKI (of any stage), with 3231 and 609 in the AKI_LOS_ and AKI_MORTALITY RISK_ subgroups, respectively (Figure 1).

Table 1 compares these 2 subgroups. In the AKI_LOS_ subgroup, the mean (±SD) age was 59.5 ± 12.2 years, 40.4% was female (n = 1306), and the mean (±SD) hospital LOS was 10.7 ± 8.4 days. In the AKI_MORTALITY RISK_ subgroup, the mean (±SD) age was 59.1 ± 12.5 years, 39.9% of patients were female (n = 243), and the mortality rate was 37.0%. The proportion of cases where AKI was diagnosed within 1 day of hospital admission was 34.2% (n = 1106) in the AKI_LOS_ subgroup and 59.3% (n = 361) in the AKI_MORTALITY RISK_ subgroup. In the AKI_MORTALITY RISK_ subgroup, 52.2% (n = 318) of cases were recorded to have at least 1 RRT session during the visit. Additional patient and hospital characteristics are shown in Table A3.

### Timely Albumin Infusion in AKI

Albumin was infused in 59.7% of cases in the AKI_LOS_ subgroup at a median dose of 20.6 g/d and in 77.8% of cases in the AKI_MORTALITY RISK_ subgroup at a median dose of 17.6 g/d, and “timely albumin” (within ≤24 hours of admission) was administered in 25.9% (n = 838) of the AKI_LOS_ subgroup and 35.8% (n = 218) of the AKI_MORTALITY RISK_ subgroup. As shown in Table 2, there were significant differences in baseline characteristics between the exposure groups (timely vs nontimely albumin administration). Patients in the timely albumin group were on average older, male, and African American and were more likely to be commercially insured. These patients also had higher MELD-Na scores, suggesting that they were sicker upon admission. Figures A1 and A2 show that patients with higher baseline MELD-Na, bilirubin, and creatinine scores were more likely to have received albumin in a timely fashion than patients with lower levels for each of these laboratory values. Additional patient and hospital characteristics are shown in Table A4.

### Association of Timely Albumin Administration With LOS (AKI_LOS_ Subgroup)

**Figure 2:**
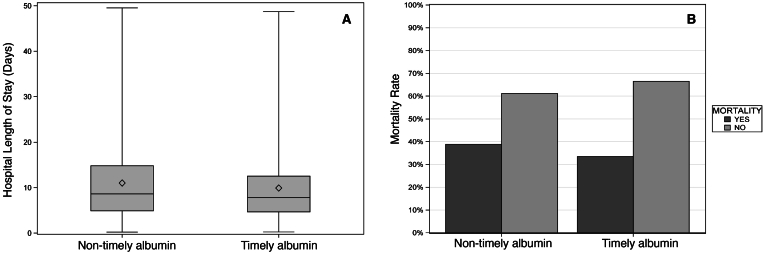
Unadjusted LOS and mortality rates by albumin group. (A) Compares treatment groups (timely vs nontimely albumin) to unadjusted hospital LOS in days; the diamond inside box represents mean, the horizontal line in the box indicates median, and the upper and lower margins of the box indicate the 25th and 75th percentiles, respectively; whiskers represent range. (B) Compares treatment groups (timely vs nontimely albumin) to unadjusted mortality rates.

The unadjusted LOS was lower in patients receiving timely albumin than in those receiving nontimely albumin (mean ± SD, 9.9 ± 7.8 vs 11.0 ± 8.6 days) (Figure 2A). Univariate analyses showed that AKI stages 2 and 3 were associated with a 5.6% increase in LOS compared to stage 1 AKI (*P* = .0661).

However, patients receiving timely albumin had a higher baseline MELD-Na and increased rates of mechanical ventilation and antibiotic receipt as compared to the nontimely group (Table 2). Conversely, vasopressor use (and duration of use) was lower (and shorter) in patients who were exposed to treatment with timely albumin. Table 3 summarizes results of the multivariable analysis. After adjusting for relevant covariates, including age, sex, ethnicity, geographic region/urbanicity, bed size, teaching status of the hospital, admission type, and calendar year, timely albumin was associated with a 15.5% shorter LOS (12.0 ± 0.7 days vs 14.1 ± 0.9 days) relative to the nontimely group (95% CI: −20.8% to −9.8%; *P* < .0001). Interestingly, AKI severity and MELD-Na score were not significantly associated with LOS. A lower proportion of patients who received timely albumin were discharged to a skilled nursing facility, compared to the nontimely albumin group. As expected, LOS was prolonged in patients with SBP, mechanical ventilation, antibiotics, and hyperglycemia and/or hypoglycemia.

**Table 3:** Multivariable Predictors of LOS

Covariates	LOS percent changes (95% confidence limits)
Timely vs nontimely	−15.5% (−20.8%, −9.8%)^c^
AKI stages 2 and 3 vs AKI stage 1	4.3% (−1.9%, 11.0%)
Gastroenteritis bleeding	7.2% (−0.3%, 15.1%)
Hepatic encephalopathy	1.3% (−4.5%, 7.5%)
Spontaneous bacterial peritonitis	13.1% (6.3%, 20.4%)^c^
Mechanical ventilation	48.5% (36.0%, 62.2%)^c^
Antibiotics	44.6% (35.6%, 54.1%)^c^
Nonselective beta-blocker	0.2% (−4.2%, 4.8%)
MELD-Na: 15–<20^a^	0.1% (−7.8%, 8.8%)
MELD-Na: 20–<30^a^	3.6% (−2.7%, 10.4%)
MELD > 30^a^	0.8% (−8.3%, 11%)
Hyperglycemia	19.1% (13.3%, 25.3%)^c^
Hypoglycemia	16.1% (0.9%, 33.5%)^b^
Hypoglycemia and hyperglycemia	53.4% (38.7%, 69.7%)^c^

Additional covariates included in the generalized linear model: age, sex, ethnicity, geographic region and urbanicity, bed size, and teaching status of the hospital, admission type, and calendar year.

a Using MELD-Na <15 as reference.

b *P* < .05.

c *P* ≤ .0001.

### Association of Albumin Infusion Timing With In-Hospital Mortality (AKI_MORTALITY RISK_ Subgroup)

In the unadjusted analysis of patients in the AKI_MORTALITY RISK_ subgroup, those who received timely albumin had a lower rate of death than those who did not (33.5% vs 38.9%) (Figure 2B). As in the AKI_LOS_ subgroup, timely albumin administration was associated with a lower proportion of patients requiring the vasopressor therapy in the AKI_MORTALITY RISK_ subgroup (32.6%, n = 71, vs 38.6%, n = 151) (Table 2). Among those who received vasopressors, the duration of therapy was shorter in the timely albumin group vs that in the nontimely group (mean ± SD hours, 73.0 ± 115.4 vs 102.9 ± 144.9).

**Figure 3:**
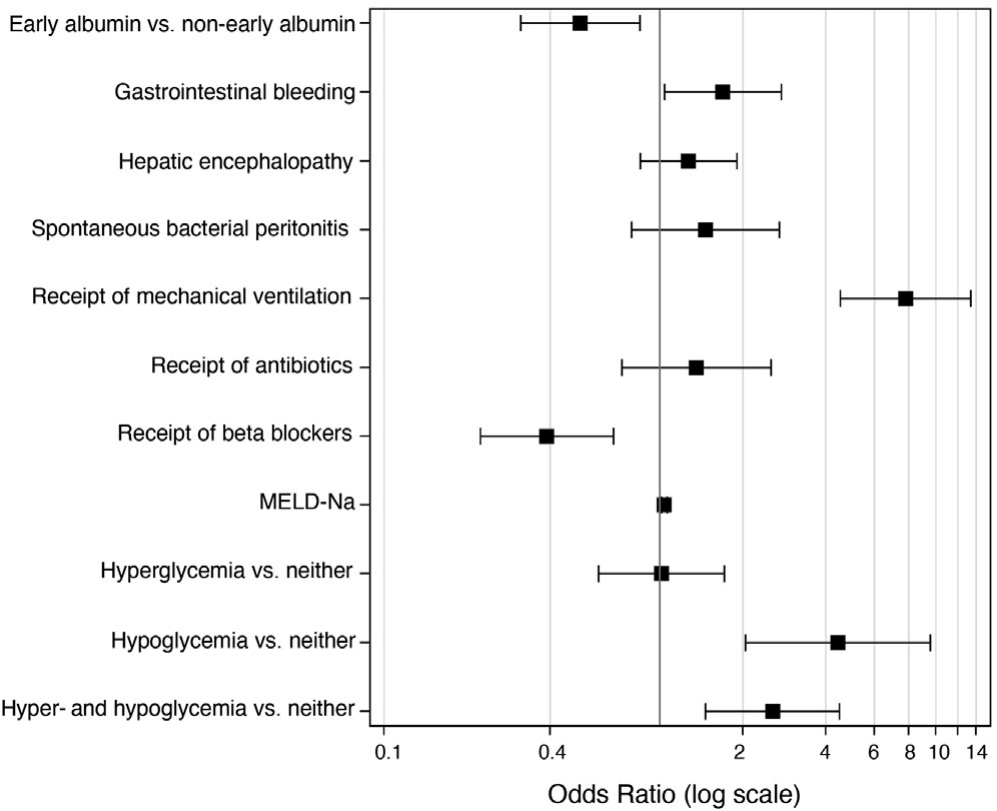
Mortality predictors vs corresponding odds ratios. A forest plot showing adjusted odds ratios of clinical characteristics and process variables predicting in-hospital mortality in the AKI_MORTALITY RISK_ subgroup. Error bars represent 95% confidence intervals. Statistically significant predictors of mortality include early albumin administration, receipt of beta-blockers, mechanical ventilation, the presence of both hypoglycemia and hyperglycemia, and isolated hypoglycemia. MELD-Na, model for end-stage liver disease.

Figure 3 summarizes the results of multivariable logistic regression. As expected, cirrhotic AKI encounters accompanied by GI bleeding, mechanical ventilation, and hypoglycemia were associated with increased mortality. Compared to those who received nontimely albumin, the odds of death were 49% lower for patients exposed to timely albumin (odds ratio: 0.51; 95% CI: 0.31–0.85; *P* = .0095) and 61% lower for patients who received beta-blockers (odds ratio: 0.39; 95% CI: 0.22–0.68; *P* < .0001).

## Discussion

AKI is a common major complication during hospitalization for decompensated cirrhosis and is associated with both increased LOS and greater mortality.^14^^,^^16^^,^^19^^,^^23^ In patients with cirrhosis who developed AKI during their hospital encounter, we found timely albumin to be associated with a shorter hospital LOS despite greater severity of illness at presentation (ie, higher MELD-Na scores at baseline), when compared to patients who did not receive timely albumin. Furthermore, among the subgroup of patients with a higher risk of death (with stage 3 AKI or RRT), timely albumin administration was associated with a 49% reduction in the odds of in-hospital mortality vs nontimely albumin. This was after adjustment for relevant covariates including common complications of cirrhosis (eg, GI bleeding, SBP, and hepatic encephalopathy). Interestingly, MELD and AKI were not associated with LOS. We suspect that this may be because the study population comprises patients who are quite ill. For example, AKI patients are expected to have consistently high SCr levels, reducing the variability in the MELD score and its detectable impact on the LOS. It is acknowledged that in these high-acuity patients, random events that adversely affect patient outcomes occur, regardless of the status of their liver morbidity at the time of presentation.

Our study has several implications for clinicians. First, in line with several prior studies, we find that the use of albumin early during hospitalization may improve patient outcomes.^27^, ^28^, ^29^, ^30^ The lowered LOS may result in reduced *overall* health-care costs and resource utilization. Cost-effectiveness has also been previously reported.^40^^,^^41^ However, among 60% of the 3231 cases where albumin was administered during the hospital encounter, the majority received albumin later during the hospital stay. Only a minority of those patients received albumin early. These results suggest that clinicians willing to use albumin should consider administration early rather than as a rescue therapy when the patients' condition worsens and/or prior therapies fail. Interestingly, in our AKI_LOS_ subgroup, 34.3% of cases developed AKI within day 1 of admission, and in the AKI _MORTALITY RISK_ subgroup, 59.3% of cases (361 out of 609) showed AKI development within day 1 of admission, suggesting that perhaps timely albumin could be protective in this patient population, irrespective of when AKI develops. The reduction in LOS could also be explained by the quality of providers who use albumin more appropriately, as sicker patients (on admission) tended to have a shorter LOS.

Second, while the general indications for albumin use during acute complications of ESLD are well established, apparent benefits of early use of albumin in patients with AKI must be interpreted in the context of recent major trials, all of which reported that mortality did not increase with albumin use. In the open label randomized trial evaluating long-term albumin administration in decompensated cirrhosis (ANSWER), patients received either standard medical treatment with albumin or standard medical treatment alone.^42^ Results showed that patients who received albumin experienced, on average, lower rates of liver-related hospitalizations as well as shorter LOS.^42^^,^^43^ Additional prospective studies that have been conducted include a randomized placebo-controlled trial which evaluated midodrine vs albumin for prevention of complications in patients with cirrhosis awaiting liver transplantation (MACHT) and a randomized trial of albumin infusions in hospitalized patients with cirrhosis (ATTIRE).^44^^,^^45^ The ATTIRE trial randomized patients with decompensated cirrhosis and hypoalbuminemia to standard medical therapy or to the additional daily administration of 20% albumin to reach a target level of 30 g/L.^45^ Neither MACHT nor ATTIRE showed a benefit compared to standard medical care, but mortality was not increased. The differences between these results and our study could be due to disparities in the study design, including dissimilar patient characteristics (eg, proportion of patients awaiting liver transplantation), dosing of albumin (weekly vs biweekly), and amount of albumin infused. Specifically, we focused on patients with a higher acuity compared to those in the ATTIRE trial (which included only ∼10% with SCr levels >1.5 mg/dL). Furthermore, approximately one-half of the ATTIRE trial’s standard treatment arm received albumin during hospitalization for complications such as SBP and hepatorenal syndrome because withholding albumin in such cases would be contrary to management guidelines.^31^^,^^32^^,^^45^ Thus, there is probable heterogeneity of treatment effects, such that early albumin infusion does not reduce the risk of mortality in patients with lower acuity. Further subgroup analyses in the context of cirrhosis should be conducted to clarify the use of albumin in various segments of patients with cirrhosis.

Our study has several limitations. First, unobserved confounding factors remain a threat to internal validity and preclude causal inference. Although we adjust for differences in the underlying severity of acute illness, it is possible that treatments other than early albumin administration explain our observation. Second, we defined exposure to albumin as timely based on its use ≤24 hours from hospital admission rather than from AKI onset. It is not clear whether albumin can be beneficial when used even earlier after the recognition of AKI. Moreover, it is possible that AKI developed on the day of admission and albumin administration on day 2 would have been considered untimely. Third, although this Cerner Electronic health record database includes granular details such as laboratory records, we excluded a substantial proportion of patients for whom there was no record of receiving fluid resuscitation. These cases are most likely to be “false negatives,” ie, patients did receive fluid therapy but without documentation. Additionally, our definition of AKI is distinct from the conventional definition in that we included baseline SCr values from the 3 months prior to admission, rather than using the typical time constraint of 48 hours.^35^^,^^36^ Using the lowest value from 3 months rather than 48 hours avoided inaccurate classification of the large number of patients that have AKI on presentation to the hospital. This approach has been shown to increase the discriminatory power in database studies of AKI.^35^ Lastly, given that the goal of this analysis was to report adherence to the guideline recommendations on albumin use with meaningful endpoints such as mortality and hospital LOS, we did not extensively explore laboratory data (other than SCr) such as serum albumin or physiological parameters such as mean arterial pressure. Although such data points would serve as interesting additions to future research in the context of cirrhosis and AKI, we posit that they are unlikely to alter the main finding of our study that timely albumin administration improves patient outcomes. Despite these limitations, our findings are biologically plausible, the temporality is clear, we have adjusted for measurable confounding, and the strength of the association comes from a robust sample size representative of the commercially insured US patient population.

## Conclusion

In conclusion, our study highlights the potential benefits of prompt albumin use in patients with decompensated cirrhosis and acute renal dysfunction. These results demonstrate the potential for timely albumin exposure to reduce LOS and in-hospital mortality, complementing recent clinical trials and studies showing the economic benefits of albumin. However, there remains a need for large, randomized trials concerning the use of albumin in patients with decompensated cirrhosis and AKI.

## Supplementary Material

Tables A1–A5, Figures A1 and A2
