# Supplementary material for: Timely Albumin Improves Survival in Patients With Cirrhosis on Diuretic Therapy Who Develop Acute Kidney Injury: Real-World Evidence in the United States
Source: Gastro Hep Adv. 2022 Oct 26;2(2):252–60. doi: 10.1016/j.gastha.2022.10.008 (PMC11307587; doi:10.1016/j.gastha.2022.10.008)
Supplement: Tables A1–A5, Figures A1 and A2 [file mmc1.docx]

**Supplementary Materials**

**Title:** Timely albumin improves survival in patients with cirrhosis on diuretic therapy who develop acute kidney injury: Real-world evidence in the United States

**Authors:** W. Ray Kim, MD; Karthik Raghunathan, MD, MPH; Greg S. Martin, MD, MSc; E. Anne Davis, PharmD, MS; Navreet Sandhu Sindhwani, MD; Santosh Telang, MS; Kunal Lodaya, MD

**Supplement Table of Contents**

Supplemental Table 1: ICD codes & descriptions for cirrhotic complications & procedures Page 3

Supplemental Table 3: Lab procedures for serum creatinine and glucose levels Page 4

Supplemental Table 4: Additional patient/hospital characteristics by AKI subgroup Page 5

Supplemental Table 5: Additional patient/hospital characteristics by albumin infusion timing

Page 6

Supplemental Figure 1: Percent of visits with albumin infusion vs. unadjusted MELD-Na Page 7

Supplemental Figure 2: Time to albumin infusion (in days) vs. unadjusted mean MELD-Na Page 8

**Supplemental Table 1: ICD codes and descriptions for cirrhotic complications and procedures**

| **Complication / Procedure** | **Type** | **Code** | **Description** |
| --- | --- | --- | --- |
| RRT | ICD-9-CM | 39.95 | Hemodialysis |
|  | ICD-9-CM | 54.98 | Peritoneal dialysis |
|  | ICD-9-CM | 38.95 | Venous catheterization for renal dialysis |
|  | ICD-10-CM | 5A1D00Z | Performance of urinary filtration, single |
|  | ICD-10-CM | 5A1D60Z | Performance of urinary filtration, multiple |
|  | ICD-10-PCS | 3E1M39Z | Irrigation of Peritoneal Cavity using Dialysate, Percutaneous Approach |
| LVP | ICD-9-CM | 54.91 | Percutaneous abdominal drainage |
|  | ICD-10-PCS | 0W9G3ZZ | Drainage of Peritoneal Cavity, Percutaneous Approach |
| SBP | ICD-9-CM | 567.23 | Spontaneous bacterial peritonitis |
|  | ICD-10-CM | K65.2 | Spontaneous bacterial peritonitis |
| HE | ICD-9-CM | 572.2 | Hepatic encephalopathy |
|  | ICD-10-CM | K72.90 | Hepatic failure, unspecified without coma |
|  | ICD-10-CM | K72.91 | Hepatic failure, unspecified with coma |
|  | ICD-10-CM | K70.41 | Alcoholic hepatic failure with coma |
|  | ICD-10-CM | K72.01 | Acute and subacute hepatic failure with coma |
|  | ICD-10-CM | K72.11 | Chronic hepatic failure with coma |
| GI Bleeding | ICD-9-CM | 456.0 | Esophageal varices with bleeding |
|  | ICD-9-CM | 530.82 | Esophageal hemorrhage |
|  | ICD-9-CM | 531.40 | Chronic stomach ulcer with bleeding |
|  | ICD-9-CM | 532.00 | Acute bleeding duodenal ulcer |
|  | ICD-9-CM | 578.0 | Hematemesis |
|  | ICD-9-CM | 578.1 | Blood in stool |
|  | ICD-9-CM | 578.9 | Hemorrhage of gastrointestinal tract, unspecified |
|  | ICD-10-CM | I85.01 | Esophageal varices with bleeding |
|  | ICD-10-CM | K92.0 | Hematemesis |
|  | ICD-10-CM | K92.1 | Melena |
|  | ICD-10-CM | K92.2 | Gastrointestinal hemorrhage, unspecified |
| Mechanical Ventilation | ICD-9-CM | 93.90 | Non-Invasive Mechanical Ventilation |
|  | ICD-10-PCS | 5A09357 | Assistance with Respiratory Ventilation, Less than 24 Consecutive Hours, Continuous Positive Airway Pressure |
|  | ICD-10-PCS | 5A09457 | Assistance with Respiratory Ventilation, 24-96 Consecutive Hours, Continuous Positive Airway Pressure |
|  | ICD-10-PCS | 5A09557 | Assistance with Respiratory Ventilation, Greater than 96 Consecutive Hours, Continuous Positive Airway Pressure |
|  | ICD-9-CM | 96.70 | Continuous Invasive Mechanical Ventilation of Unspecified Duration |
|  | ICD-9-CM | 96.71 | Continuous Invasive Mechanical Ventilation for Less Than 96 Consecutive Hours |
|  | ICD-9-CM | 96.72 | Continuous Invasive Mechanical Ventilation for 96 Consecutive Hours or More |
|  | ICD-10-PCS | 5A1935Z | Respiratory Ventilation, Less than 24 Consecutive Hours |
|  | ICD-10-PCS | 5A1945Z | Respiratory Ventilation, 24-96 Consecutive Hours |
|  | ICD-10-PCS | 5A1955Z | Respiratory Ventilation, Greater than 96 Consecutive Hours |

**Supplemental Table 2: Lab procedures for serum creatinine and glucose levels**

| **Lab Procedure Name** | **Lab Procedure Group** | **LOINC Code** |
| --- | --- | --- |
| Creatinine, Serum Quantitative | Creatinine Test | 2160-0 |
| Creatinine, Serum 4 hr. |  | 16189-3 |
| Creatinine, Whole Blood ISTAT |  | 59826-8 |
| Creatinine, Whole Blood NOVA |  | 38483-4 |
| Glucose, Serum/Plasma Quantitative | Glucose Test | 2345-7 |
| Glucose Blood |  | 5914-7 |
| Glucose Stick/Meter Whole Blood POC |  | 2339-0 |
| Glucose, Serum 2 hr pp |  | 1521-4 |
| Glucose, Serum Fasting |  | 14771-0 |
| Glucose, Serum Random |  | 2345-7 |
| Glucose, Whole Blood GMD |  | 2339-0 |
| Glucose, Whole Blood Quantitative |  | 2339-0 |
| Glucose, Whole Blood Prec G Random |  | 2339-0 |
| Glucose, Whole Blood Random |  | 2339-0 |
| Glucose, Whole Blood Random Nova |  | 2339-0 |
| Glucose, Whole Blood dup |  | 2339-0 |
| Glucose, Serum 1 hr pp |  | 10449-7 |
| Glucose, Serum 15 min |  | 12639-1 |
| Glucose, Serum 30 min |  | 40042-4 |
| Glucose, Serum 45 min |  | 21309-0 |
| Glucose, Serum 90 min |  | 40003-6 |
| Glucose, Quantitative Capillary Blood, Glucometer |  | 41653-7 |
| Glucose, Serum/Plasma Fasting, Quantitative |  | 1558-6 |
| Glucose Mass/Vol Blood, Automated Test Strip |  | 2340-8 |
| Glucose Mass/Vol Blood, Test Strip, Manual |  | 2341-6 |
| Glucose Moles/Vol Blood, Automated Test Strip |  | 72516-8 |
| Glucose Mass/volume Blood, Automated Test Strip |  | 2340-8 |
| Glucose Mass/volume Blood, Test Strip, manual |  | 2341-6 |
| Glucose Mass/volume Arterial Blood |  | 41651-1 |
| Glucose Moles/volume Arterial Blood |  | 39481-7 |
| Glucose Mass/volume Venous Blood |  | 41652-9 |
| Glucose Moles/volume Venous Blood |  | 39480-9 |
| Glucose Moles/volume Capillary Blood |  | 51596-5 |
| Glucose Moles/volume Capillary Blood, glucometer |  | 14743-9 |

**Supplementary Table 3: Additional patient/hospital characteristics by AKI group**

|  | **All AKI**  **(N=4,135)** | **AKI_LOS_^1^**  **(N=3,231)** | **AKI_mortality_**  **(N=609)** |
| --- | --- | --- | --- |
| Index year, n (%) | | | |
| 2009 | 114 (2.8) | 95 (2.9) | 16 (2.6) |
| 2010 | 190 (4.6) | 151 (4.7) | 28 (4.6) |
| 2011 | 217 (5.3) | 166 (5.1) | 29 (4.8) |
| 2012 | 306 (7.4) | 225 (7.0) | 30 (4.9) |
| 2013 | 478 (11.6) | 356 (11.0) | 68 (11.2) |
| 2014 | 782 (18.9) | 624 (19.3) | 135 (22.2) |
| 2015 | 561 (13.6) | 428 (13.3) | 91 (14.9) |
| 2016 | 626 (15.1) | 499 (15.4) | 98 (16.1) |
| 2017 | 631 (15.3) | 502 (15.5) | 86 (14.1) |
| 2018 | 230 (5.6) | 185 (5.7) | 28 (4.6) |
| Charlson comorbidities, n (%) | | | |
| Myocardial infarction | 596 (14.4) | 469 (14.5) | 102 (16.8) |
| Congestive heart failure | 1,480 (35.8) | 1,170 (36.2) | 246 (40.4) |
| Peripheral vascular disease | 593 (14.3) | 480 (14.9) | 102 (16.8) |
| Cerebrovascular disease | 452 (10.9) | 348 (10.8) | 77 (12.6) |
| Dementia | 104 (2.5) | 78 (2.4) | 14 (2.3) |
| Chronic pulmonary disease | 1,621 (39.2) | 1,283 (39.7) | 244 (40.1) |
| Rheumatic disease | 197 (4.8) | 157 (4.9) | 22 (3.6) |
| Peptic ulcer disease | 437 (10.6) | 351 (10.9) | 78 (12.8) |
| Mild liver disease | 846 (20.5) | 692 (21.4) | 104 (17.1) |
| Diabetes without chronic complications | 1,101 (26.6) | 870 (27.0) | 144 (23.7) |
| Diabetes with chronic complications | 749 (18.1) | 634 (19.6) | 143 (23.5) |
| Hemiplegia or paraplegia | 87 (2.1) | 64 (2.0) | 16 (2.6) |
| Renal disease | 1,790 (43.3) | 1,427 (44.2) | 338 (55.5) |
| Malignant cancer^2^ | 468 (11.3) | 353 (10.9) | 61 (10.0) |
| Moderate or severe liver disease | 2,937 (71.0) | 2,274 (70.4) | 455 (74.7) |
| Metastatic solid tumor | 312 (7.6) | 220 (6.8) | 52 (8.5) |
| Acquired Immune Deficiency Syndrome/HIV | 69 (1.7) | 55 (1.7) | 15 (2.5) |
| Discharge status, n (%) |  |  |  |
| Death | 895 (21.6) | - | 225 (37.0) |
| Home | 1,693 (40.9) | 1,689 (52.3) | 177 (29.2) |
| Hospice | 393 (9.5) | 392 (12.1) | 50 (8.2) |
| Inpatient Hospital | 67 (1.6) | 67 (2.1) | 6 (1.0) |
| Other Post-Acute Care Setting | 337 (8.2) | 336 (10.4) | 52 (8.5) |
| Skilled Nursing Facility | 500 (12.1) | 500 (15.5) | 53 (8.7) |
| Not Specified | 250 (6.1) | 247 (7.6) | 46 (7.6) |
| Bed size, n (%) | | | |
| <100 | 279 (6.8) | 221 (6.8) | 33 (5.4) |
| 100-199 | 721 (17.4) | 592 (18.3) | 113 (18.6) |
| 200-299 | 844 (20.4) | 682 (21.1) | 112 (18.4) |
| 300-499 | 1,130 (27.3) | 869 (26.9) | 165 (27.1) |
| 500+ | 1,161 (28.1) | 867 (26.8) | 186 (30.5) |
| Teaching facility, n (%) | 2,904 (70.2) | 2,224 (68.8) | 407 (66.8) |
| Hospital type, n (%) | | | |
| Urban | 3,392 (82.0) | 2,663 (82.4) | 446 (76.5) |
| Rural | 743 (18.0) | 568 (17.6) | 143 (23.5) |
| Census region, n (%) | | | |
| South | 1,796 (43.4) | 1,459 (45.2) | 235 (38.6) |
| Midwest | 531 (12.8) | 425 (13.2) | 90 (14.8) |
| Northeast | 1,115 (27.0) | 833 (25.8) | 153 (25.1) |
| West | 693 (16.8) | 514 (15.9) | 131 (21.5) |

^1^excludes decedents. ^2^ including lymphoma and leukemia, excluding malignant skin neoplasms.

**Supplemental Table 4: Additional patient/hospital characteristics by albumin infusion timing**

|  | **AKI_LOS_^1^ (n=3,231)** | | **AKI_mortality_ (n=609)** | |
| --- | --- | --- | --- | --- |
|  | Timely albumin (n=838) | Non-timely albumin (n= 2,393) | Timely albumin (n=218) | Non-timely albumin  (n= 391) |
| Index year, n (%) | | | | |
| 2009 | 18 (2.2) | 77 (3.2) | 1 (0.5) | 15 (3.8) |
| 2010 | 30 (3.6) | 121 (5.1) | 14 (6.4) | 14 (3.6) |
| 2011 | 41 (4.9) | 125 (5.2) | 8 (3.7) | 21 (5.4) |
| 2012 | 44 (5.3) | 181 (7.6) | 6 (2.8) | 24 (6.1) |
| 2013 | 73 (8.7) | 283 (11.8) | 20 (9.2) | 48 (12.3) |
| 2014 | 173 (20.6) | 451 (18.9) | 54 (24.8) | 81 (20.7) |
| 2015 | 126 (15.0) | 302 (12.6) | 36 (16.5) | 55 (14.1) |
| 2016 | 162 (19.3) | 337 (14.1) | 36 (16.5) | 62 (15.9) |
| 2017 | 116 (13.8) | 386 (16.1) | 28 (12.8) | 58 (14.8) |
| 2018 | 55 (6.6) | 130 (5.4) | 15 (6.9) | 13 (3.3) |
| Charlson comorbidities, n (%) | | | |  |
| Myocardial infarction |  |  |  |  |
| Congestive heart failure | 233 (27.8) | 937 (39.2) | 81 (37.2) | 165 (42.2) |
| Peripheral vascular disease | 114 (13.6) | 366 (15.3) | 40 (18.4) | 62 (15.9) |
| Cerebrovascular disease | 84 (10.0) | 264 (11.0) | 30 (13.8) | 47 (12.0) |
| Dementia | 10 (1.2) | 68 (2.8) | 5 (2.3) | 9 (2.3) |
| Chronic pulmonary disease | 279 (33.3) | 1,004 (42.0) | 84 (38.5) | 160 (40.9) |
| Rheumatic disease | 31 (3.7) | 126 (5.3) | 6 (2.8) | 16 (4.1) |
| Peptic ulcer disease | 89 (10.6) | 262 (11.0) | 32 (14.7) | 46 (11.8) |
| Mild liver disease | 125 (14.9) | 567 (23.7) | 25 (11.5) | 79 (20.2) |
| Diabetes without chronic complications | 220 (26.3) | 650 (27.2) | 43 (19.7) | 101 (25.8) |
| Diabetes with chronic complications | 145 (17.3) | 489 (20.4) | 55 (25.2) | 88 (22.5) |
| Hemiplegia or paraplegia | 13 (1.6) | 51 (2.1) | 4 (1.8) | 12 (3.1) |
| Renal disease | 378 (45.1) | 1,049 (43.8) | 119 (54.6) | 219 (56.0) |
| Malignant cancer^2^ | 101 (12.1) | 252 (10.5) | 21 (9.6) | 40 (10.2) |
| Moderate or severe liver disease | 667 (79.6) | 1,607 (67.2) | 182 (83.5) | 273 (69.8) |
| Metastatic solid tumor | 55 (6.6) | 165 (6.9) | 22 (10.1) | 30 (7.7) |
| Acquired Immune Deficiency Syndrome/HIV | 5 (0.6) | 50 (2.1) | 4 (1.8) | 11 (2.8) |
| Discharge status, n (%) | | | | |
| Death | - | - | 73 (33.5) | 152 (38.9) |
| Home | 460 (54.9) | 1,229 (51.4) | 69 (31.7) | 108 (27.6) |
| Hospice | 108 (12.9) | 284 (11.9) | 20 (9.2) | 30 (7.7) |
| Inpatient Hospital | 15 (1.8) | 52 (2.2) | 2 (0.9) | 4 (1.0) |
| Other Post-Acute Care Setting | 87 (10.4) | 249 (10.4) | 22 (10.1) | 30 (7.7) |
| Skilled Nursing Facility | 80 (10.0) | 420 (17.6) | 14 (6.4) | 39 (10.0) |
| Not Specified | 88 (10.5) | 159 (6.6) | 18 (8.3) | 28 (7.2) |
| Bed size, n (%) | | | |  |
| <100 | 36 (4.3) | 185 (7.7) | 6 (2.8) | 27 (6.9) |
| 100-199 | 161 (19.2) | 431 (18.0) | 33 (15.1) | 80 (20.5) |
| 200-299 | 154 (18.4) | 528 (22.1) | 45 (20.6) | 67 (17.1) |
| 300-499 | 218 (26.0) | 651 (27.2) | 55 (25.2) | 110 (28.1) |
| 500+ | 269 (32.1) | 598 (25.0) | 79 (36.2) | 107 (27.4) |
| Teaching facility, n (%) | 618 (73.8) | 1,606 (67.1) | 158 (72.5) | 249 (63.7) |
| Hospital type, n (%) | | | | |
| Urban | 685 (81.7) | 1,978 (82.7) | 168 (77.1) | 298 (76.2) |
| Rural | 153 (18.3) | 415 (17.3) | 50 (22.9) | 93 (23.8) |
| Census region, n (%) | | | | |
| South | 379 (45.2) | 1,080 (45.1) | 74 (33.9) | 161 (41.2) |
| Midwest | 82 (9.8) | 343 (14.3) | 31 (14.2) | 59 (15.1) |
| Northeast | 181 (21.6) | 652 (27.3 | 56 (25.7) | 97 (24.8) |
| West | 196 (23.4) | 318 (13.3) | 57 (26.2) | 74 (18.9) |

^1^excludes decedents. ^2^ including lymphoma and leukemia, excluding malignant skin neoplasms.


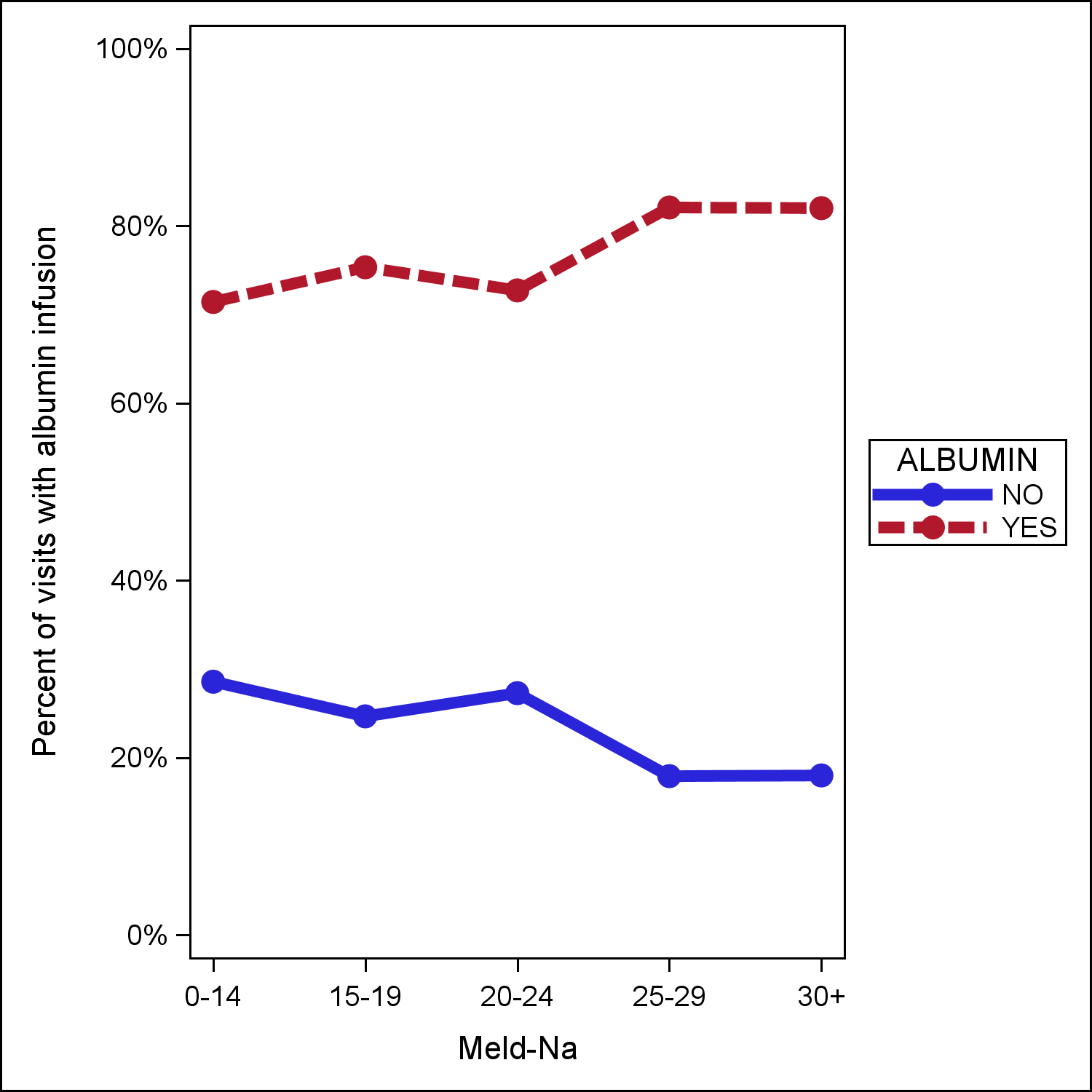

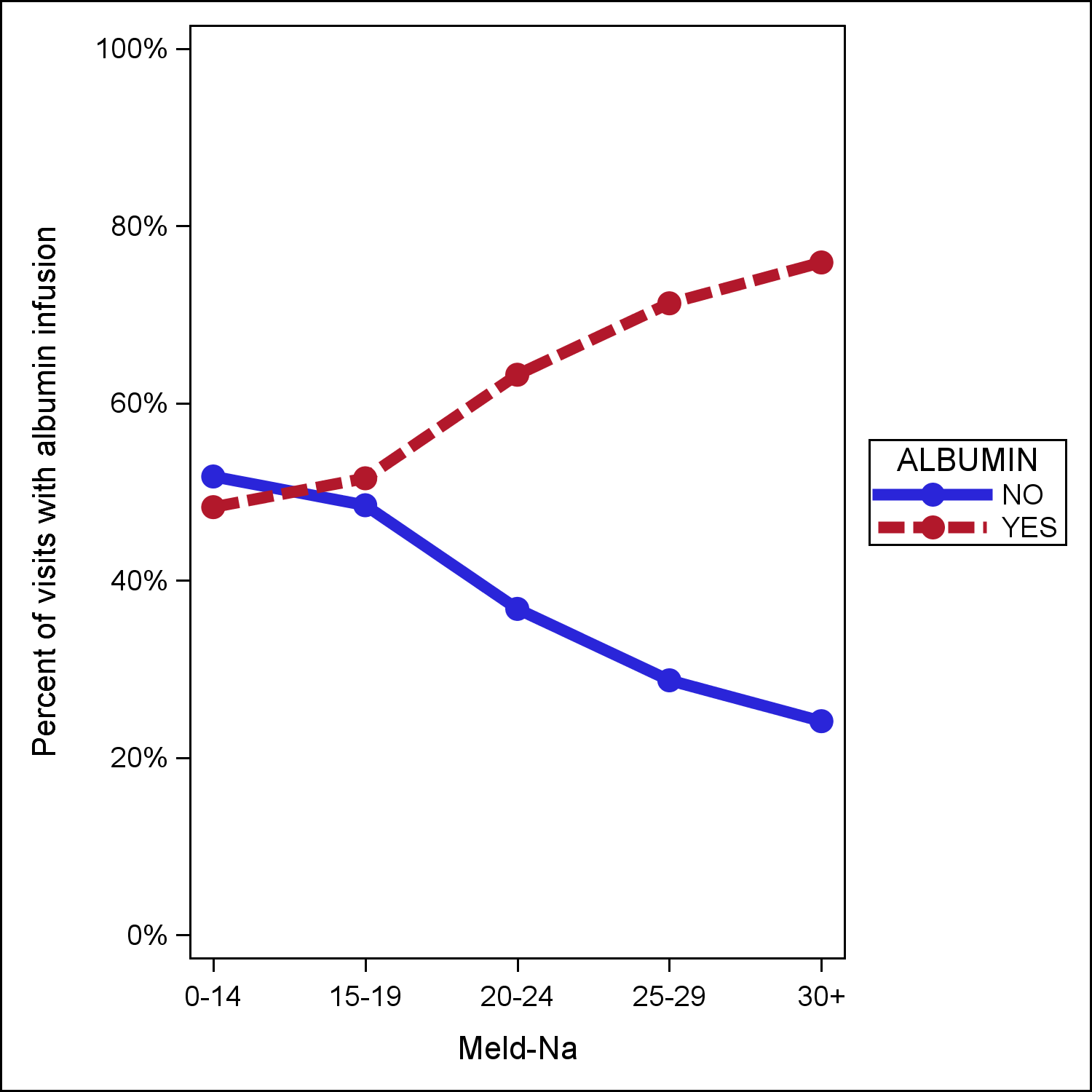


**A**

**B**

Percent of hospitalizations with albumin infusion

Percent of hospitalizations with albumin infusion

**Supplemental Figure 1: Percent of visits with albumin infusion vs. unadjusted mean MELD-Na. (A) AKI_LOS_ subgroup; (B) AKI_mortality_ subgroup.**


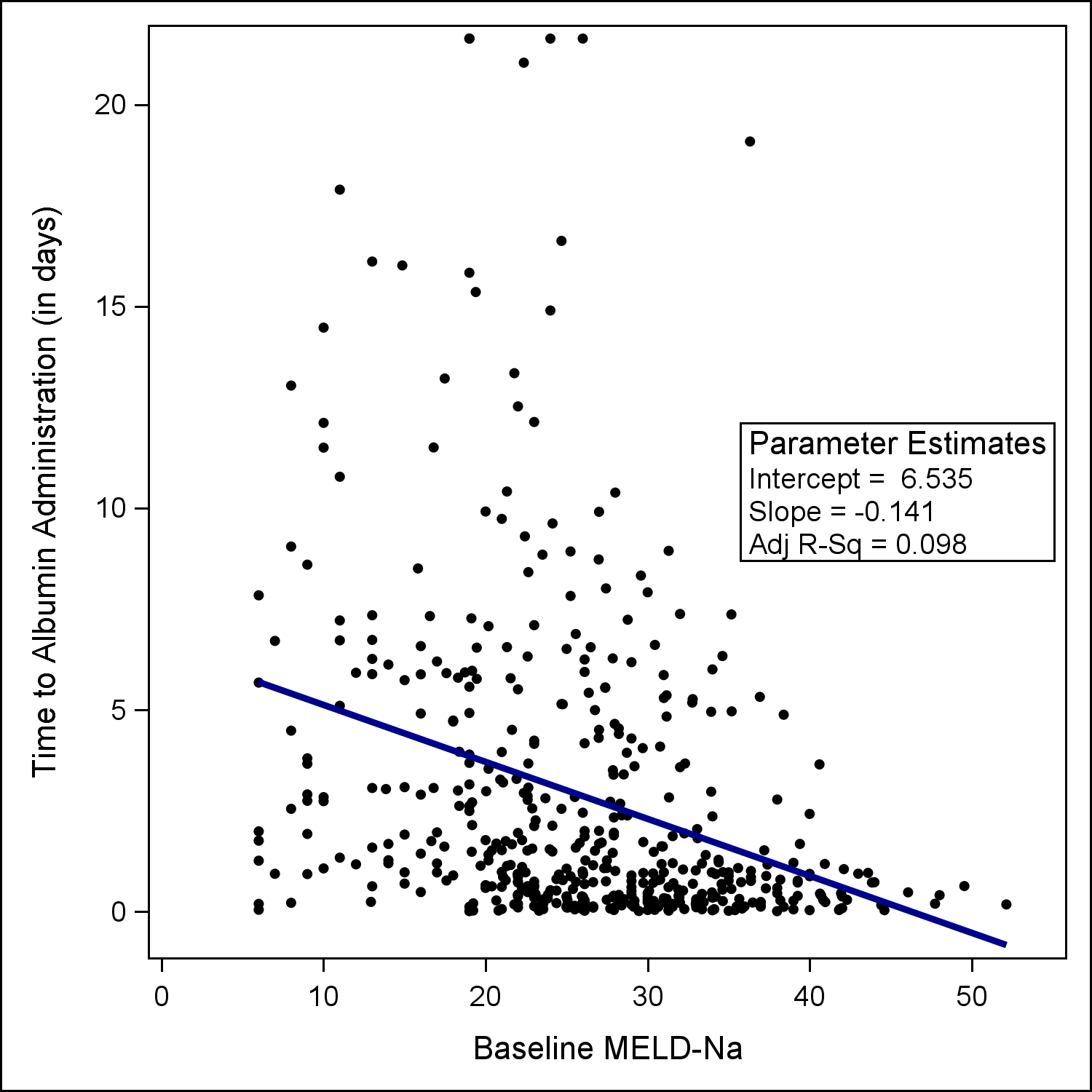

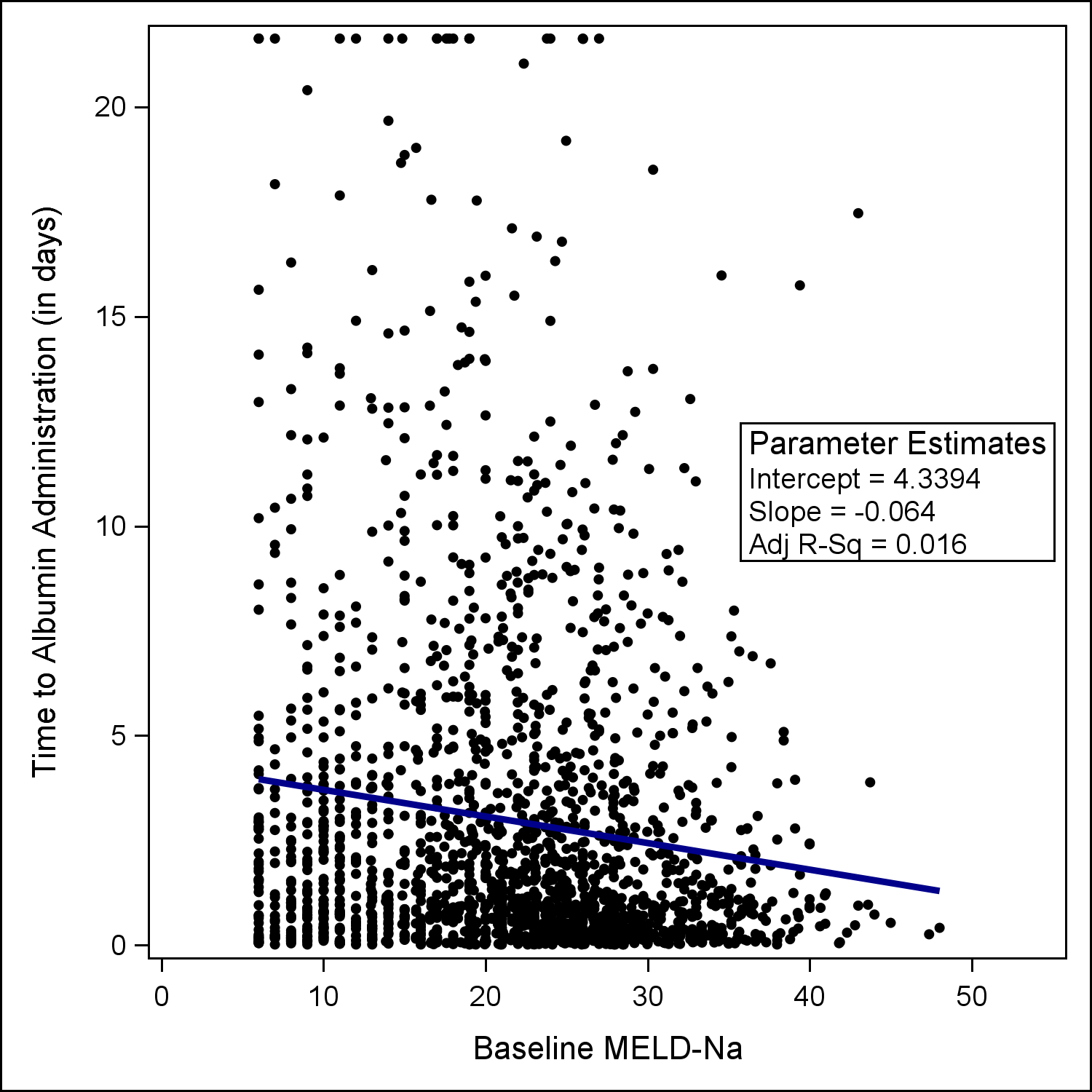


**A**

**B**

**Supplemental Figure 2: Time to albumin (in days) vs. unadjusted mean MELD-Na. (A) AKI_LOS_ subgroup; (B) AKI_mortality_ subgroup**
